# Empathy development from adolescence to adulthood and its consistency across targets

**DOI:** 10.3389/fpsyg.2022.936053

**Published:** 2022-10-10

**Authors:** Augusta Gaspar, Francisco Esteves

**Affiliations:** ^1^Católica Research Centre for Psychological—Family and Social Wellbeing, Universidade Católica Portuguesa, Lisbon, Portugal; ^2^Department of Psychology and Social Work, Mid Sweden University, Östersund, Sweden

**Keywords:** empathy, empathy development, empathy across targets, facial expression decoding, dynamic facial expression stimuli, adolescent development, animal-directed empathy, emotional development

## Abstract

This research was conducted with two main goals—to contribute to knowledge on the development of empathy from early adolescence to adulthood, including its contribution to decoding emotion expression, and to improve the understanding of the nature of empathy by simultaneously assessing empathy toward two different targets—humans and animals. It unfolded into two cross-sectional studies: One (S1) obtaining measures of empathy toward humans and animals as targets across five age groups (from pre-adolescents to adults); and another (S2) where a subset of the adolescents who participated in S1 were assessed in emotion expression decoding and subjective and physiological responses to emotional video clips. The results of S1 showed that empathy toward animals and most dimensions of empathy toward humans increase toward adulthood, with important gender differences in empathy to animals and humans, and empathy levels in girls starting off in the age trajectory at higher levels, A moderate correlation between empathy toward human and toward animal targets was also found. S2 showed that the expression of positive emotion is better recognized than that of negative emotion, surprise, or neutral expression, and that the measure of human-directed empathy predicts successful decoding of negative emotion, whereas skin conductance responses (SCRs) and subjective valence ratings predicted successful identification of positive emotion. Gender differences emerged but not across all age groups nor all subscales. Results yield keys to the developmental “pace” and trajectory of the various dimensions of empathy and to how empathy relates to emotion decoding.

## Introduction

Empathy is defined as an emotional experience in response to the perception of someone else’s emotion, and entails either the mirroring of that someone’s emotion or another affective state, motivating the individual to act—to help, relieve pain, or to partake in the other person’s positive experience, depending on the specific emotion that is perceived (Eisenberg and Miller, 1987; Hoffman, 2000). It is largely agreed that empathy comprises an affective and a cognitive component (mentalizing) (Preston and de Waal, 2002; Decety and Jackson, 2004) acknowledged even across models that entail more than two dimensions (e.g., Davis, 1983; Blair, 2005).

Empathy varies inter- individually, and as a dispositional trait it has been approached as an overarching concept whereby if one scores high in empathy he/she is said to be highly empathic, and consequently expected to respond with an equivalent high strong reaction to all *kinds* of empathy triggers. Even though trait-empathy largely predicts states of mind, behavior and interactions—ranging from the likelihood of helping someone in need, to abiding to COVID-19 safety rules (Pfattheicher et al., 2020)—there are indicators that it does not strongly predict identical empathic concern or empathic reactions toward different targets: for example, familiarity, identification, personal bond or the target being under a spotlight (Batson et al., 2005; Bloom, 2017) have shown to also play a role.

### Empathy across age groups

**The study of empathy development across the life span** still holds many open questions. With most research focused on the first years of life, there is a converging view that empathy increases from childhood to adolescence (e.g., Dymond et al., 1952; Eisenberg and Mussen, 1989) and that differences between males and females in both neural network maturation and affective and behavioral responses occur very early (Decety and Svetlova, 2012); there is also evidence of critical windows of opportunity and key influences for its development in childhood (Eisenberg and Mussen, 1987, 1989). To the contrary, empathy development throughout the adult life span has received less attention with studies providing different portrayals—from no differences across ages to a decrease toward old age, and a possible link between maintaining empathy high along adulthood, literacy, and mostly the richness of social interactions (Grühn et al., 2008).

Adolescence entails important developments in emotional experience and regulatory processes (Eisenberg, 2000; Gross, 2015) making this group a key target for the study of the development of empathy and one that may contribute to understanding the processes that take place in the trajectory from child empathy to adult empathy. Although early adolescence has been pointed as a critical period in empathy development (van Lissa et al., 2014) and adolescence as a period of crucial charges in emotion regulation (Cole and Jacobs, 2018) it is not clear if empathy follows a linear path from early to late adolescence. If empathy follows the path of other emotional experiences, then it would peak around mid to late adolescence, and empathic responses would have been slightly down-regulated toward late adolescence. A recent review indicates a trend toward more frequent and intense negative emotions in late adolescence and a decrease in the frequency of positive emotions, as well as more intense positive and negative emotions in adolescents than in adults (Bailen et al., 2019); this review was, however, limited to the few available studies, some of them conducted over 20 years ago. Adults had already been reported to score higher on empathy than adolescents (Grühn et al., 2008), but again this account is from limited studies.

The pathway of empathy development from early adolescence to adulthood yet to be fully described. There is currently no consensus on the contribution of empathy to emotion processing skills such as relating facial expression to emotional experience, and the role of various environmental influences still needs to be understood.

Hoffman, 1975, 1982, 2000 proposes that children’s prosocial behavior is influenced by parental models of behavior toward targets, not especially by means of words, but by social modeling—children form beliefs, and attitudes and engage in prosocial behavior consistent with that of their parents. So an example would be that the way parents behave toward homeless people, migrants, or animals shapes beliefs and behavior toward those targets than whatever parents might say are the adequate behaviors or attitudes. Because prosocial behavior is largely predicted by empathy, we may assume that parental behavior is key in developing empathy. But research on empathy is yet to address the question of if and how empathy toward different targets develops and if there is any synchrony in the development of empathy across targets. In adulthood, empathy toward animals is predicted by having grown up with animal pets (Paul, 2000; Emauz et al., 2018). If social modeling of behavior and attitudes is a major force in shaping empathy, perhaps then it would not be fully synchronized across targets—as many people sympathize with human suffering, but not all human suffering equally, and not all in the former group, empathize with animal suffering. Previous findings point in this direction—if empathy stems from the mother-infant bond and thus from a mechanism that generalizes to other humans (de Waal, 2008), human targeted empathy is expected to be the norm. But some people empathize more with animals than with humans (Knight and Barnett, 2008), with preliminary findings showing modest correlations of empathy toward humans and animals—ranging from 0.25 to 0.3 (Paul, 2000; Emauz et al., 2018). Nevertheless, it is not established whether in the extreme groups of empathy (very high and very low) this correlation is not substantially different, and perhaps much higher in the high empathy group, which would possibly indicate the prevalence of a disposition over an external influence, modeling, cultural values, and beliefs, or any other.

**So our first goal** was to examine the possible differences across age groups in the various dimensions of empathy and to address the question of whether empathy is congruent across different target groups (S1)—humans and other animals—as that will contribute toward disentangling empathy stemming from a deep trait root *or* from a social and exposure source such as early target exposure, social learning and conforming with group attitudes in empathy.

### Empathy in adolescents and decoding emotional expression

A large body of literature indicates the important role individual traits of the perceiver play in decoding facial expressions. Examples range from cultural representations and beliefs (e.g., Hess et al., 2002), to one’s own emotionality and expressiveness (Halberstadt et al., 2011), to sex, age, familiarity, or identification with the target (e.g., Bloom, 2017). Humans are equipped with mechanisms that facilitate pre-attentive detection of empathy-relevant stimuli (for a review see Gaspar, 2021) and an empathic reaction may be triggered without much, if any, thought. One of the reasons why such automatic empathic responses may be relevant to the detection and decoding of emotional signals is that the affective response activates the networks of one’s own experience—as implied in Preston and de Waal’s (2002) Perception action Model—and the activation of these networks becomes speedier with the accumulation of one’s own experience, and so does the conscious subjective experience and related motivated behavior. Because the affective component of empathy develops earlier and faster (Decety and Michalska, 2010) than the cognitive one, this should be a major contributor to emotion decoding in earlier years. This would not be a consensual view as other authors suggested that the perspective-taking component of empathy may be the one driving the ability to decode emotional cues (Feshbach, 1982) and the ulterior affective response, or that the success of children in facial emotion identification is key to their empathic responding (Hutman and Dapretto, 2009). The few studies on the role of empathy in people’s facial expression decoding skills indicate a relation between trait empathy and the ability to accurately detect and interpret prototypical facial expressions of “basic emotions,” measuring automatic implicit reactions (e.g., Balconi and Canavesio, 2016) and conscious appraisals (Besel and Yuille, 2010; Ávila et al., 2016). These studies were conducted with adults and there are as of now, no reports on this relation in adolescents. Additionally, affective dimensions of trait empathy have also been shown to predict accuracy in a task of determining the authenticity of laughter from playback audio (Neves et al., 2018).

So, in this paper, we also inspect (S2) whether trait empathy and its cognitive and affective dimensions, predict accuracy in the interpretation of emotional expression by adolescents.

## Study 1. Empathy through adolescence and into adulthood and is it consistent across targets?

In this study (S1) we address the question of empathy development from a comparative perspective—measuring trait empathy in preadolescents, middle adolescents, late adolescents and adults toward humans and animals to explore the possible developmental pathway and examine the congruence in empathy toward the different targets.

## Materials and methods

### Participants

Adolescent participants were recruited in three schools in the great Lisbon area and took part voluntarily in study1; only a fraction was included in study 2 because many failed to complete questionnaires or to keep their code number card, a requirement to continue in the study and retain anonymity. Adults were recruited via email, social media and announcements in academic media, and also took part on a voluntary basis. All participants signed an informed consent form prior to entering the study. All participants under 19 years old (*N* = 468), were assessed at their respective schools (in both studies), whilst adults (*N* = 149) were assessed at the Psychology Lab. Study 1 was based on the responses of 617 participants (ages 11–55, 57% girls, 43% boys) divided into five age groups 11–12 years old; 13–14 years old; 15–16 years old; 17–18 years old, and adults (19 years old and above). Female participants exceeded male participants in all but the 13–14 years old group.

The sample of participants under 19 years old was broken down into four 2-year range groups departing from a pre-adolescent group, considering the onset of puberty at about 11–12 years old, adolescence through the “teen years” with a later period beginning the transition to adulthood at 17–18 years (e.g., Smith et al., 2009), and adulthood beginning at 19 years of age. Although we had fewer volunteer participants in the younger group we thought it was important to keep it, as pooling groups (for example into early/middle and late adolescence) could blur changes beginning in those two pre-teen years—identified as the starting points of swift transitions in emotion relevant experiences likely to affect the results of our study, such as increased compassion (Cowie, 2012), self-and other regulatory competencies (e.g., Ahmed et al., 2015), changes in concerns (Erikson, 1968), alongside with changes in brain organization, affecting the top-down control of emotional reactivity (Young et al., 2019), and a diversity of cognitive functions and behavior (e.g., Berenbaum and Beltz, 2011). Regarding the adult group, the literature diverges as to the onset of adulthood, with some authors incorporating 19-year-old youth in adolescence (e.g., Salmela-Aro, 2011) and others considering it the beginning of adulthood (e.g., Hare et al., 2008; Tottenham et al., 2011); as our 19-year-old participants were respondents of the online survey along with other adults, it only made sense to include them in this group. Table 1 provides detailed information on the five age groups according to gender.

**TABLE 1:** Characterization of participants in S1 according to five age groups and gender.

	Gender					
	Female participants (*N* = 351)			Male participants (*N* = 266)		
Age groups (years)	Total	Mean	*SD*	Total	Mean	*SD*
11–12	29	11.93	0.25	17	12	0.00
13–14	83	13.39	0.50	87	13.55	0.50
15–16	81	15.36	0.48	61	15.39	0.49
17–18	68	17.4	0.50	42	17.33	0.47
19–55	90	22.81	5.71	59	24.86	7.88

### Measures

#### Measure of empathy toward humans

The Portuguese version (Limpo et al., 2010) of the *Interpersonal Reactivity Index—IRI* (Davis, 1980, 1983) is comprised of four scales, one that captures more cognitive components of empathy (the Perspective Taking scale), two that capture one’s affective typical response regarding other’s negative experiences (the Personal distress and Empathic concern scales), and the Fantasy scale, capturing one’s tendency to identify with characters in movies, novels, plays, and other fictional media—so, in a sense, also entailing an affective component. Participants respond to 24 items on a five-point scale from 0 (does not describe me well) to 4 (describes me very well).

#### Measure of empathy toward animals

The Portuguese version of the *Animal Empathy Scale—AES* (Emauz et al., 2016) that was developed to assess animal-oriented trait empathy, with a strong emphasis on the emotional component of empathy (Paul, 2000). The Portuguese version entails two dimensions instead of the single one in the original version, one entailing an Emotional Connection with Animals and the other capturing Empathic Concern with animals. Participants respond to 13 items on a nine-point scale from 1 (I fully disagree) to 9 (I fully agree).

### Procedures

Adolescent participants were recruited at the school grounds on a volunteer basis and after school consent was provided. They filled out the questionnaires at school and delivered them to a research team member. Each participant received a code number to ensure anonymity and to be identified as a possible participant in study 2 and signed informed consent prior to filling out the questionnaire. Adults filled the questionnaires at the university psychology facilities upon signing informed consent. They responded to a call for volunteer participants as part of another study not reported here and received a voucher after participation in the study.

## Results

### The development of empathy and empathy through adolescence and into adulthood

**FIGURE 1:**
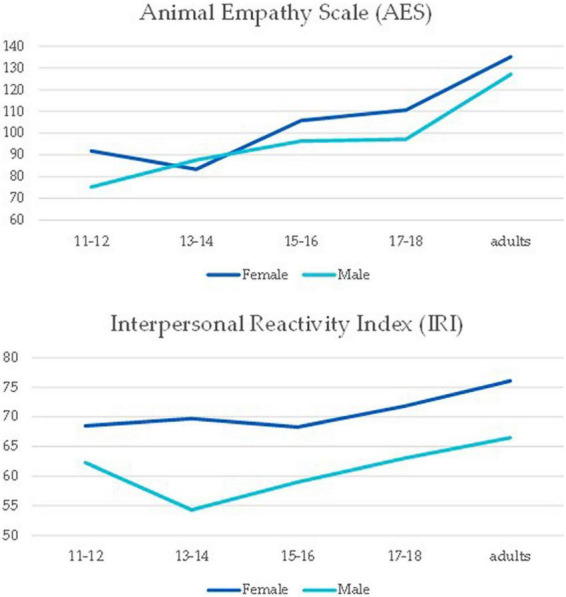
Mean values in the Animal Empathy Scale (AES) (top), and in the Interpersonal Reactivity Index (IRI) (bottom), for female and male participants, in the five age groups.

Between groups 2 × 5 ANOVAs show that the five age groups vary significantly, with a general increase with age, in both their IRI scores, *F*(4, 607) = 13.05, *p* < 0.001, η^2^ = 0.07, and their AES scores, *F*(4, 607) = 51.16, *p* < 0.001, η^2^ = 0.25. Gender differences were also evident, with female participants presenting higher empathy scores, both in IRI, *F*(1, 607) = 79.14, *p* < 0.001, η^2^ = 0.11, and in AES, *F*(1, 607) = 9.95, *p* = 0.002, η^2^ = 0.01 (see Figure 1). The interaction between gender and age group was marginally significant for IRI, *F*(4, 607) 2.36, *p* = 0.05, η^2^ = 0.01, but not for AES, *F*(4, 607) = 2.12, *p* = 0.08, η^2^ = 0.01.

**FIGURE 2:**
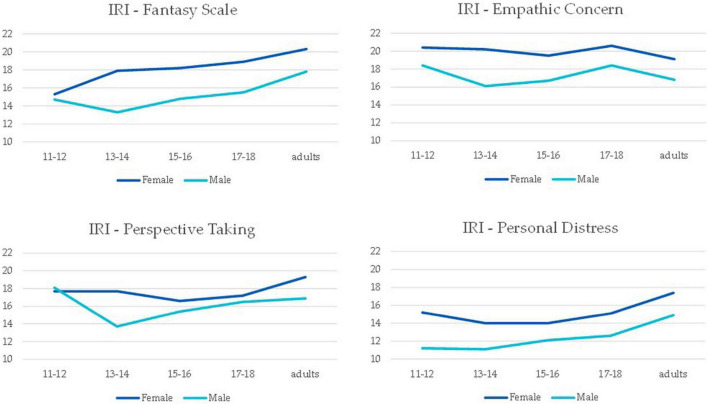
Mean values in the four sub-scales of the Interpersonal Reactivity Index (IRI) for female and male participants, in the five age groups.

Analyzing the four subscales of IRI, the general pattern was similar regarding gender (all *F* > 11.31, *p* < 0.001), however, there were some interesting differences regarding the development of empathy through age. While the increasing relationship was observed for the IRI-FS and IRI-PD, no age differences were observed for the IRI-EC subscale, *F*(4, 607) 2.13, *p* = 0.08, η^2^ = 0.01. Regarding IRI-PT an interaction was observed, *F*(4, 607) 2.91, *p* = 0.02, η^2^ = 0.02. As it can be seen in Figure 2, it is in the age group 13–14 that the difference is significant, *p*_*tukey*_ < 0.001.

To better understand the impact of age and gender on the empathy scores, we tested a regression model with age and gender as predictors with AES, IRI, and each of IRI’s subscales as dependent variables, running a total of 6 analyzes. The summarized results are presented in Table 2.

**TABLE 2:** The role of age and gender in the prediction of the self-report empathy measures in study 1, as assessed with a linear regression model.

			B (SE)	β	*t*	B (SE)	*t*
Dependent variable	Model adjusted *R*^2^	Model *F*	(Age)	(Age)	(Age)	(Gender)	(Gender)
AES	0.23***	93.07	0.24 (0.47)***	0.19	13.18	−8.81 (2.50)***	3.55
IRI	0.20***	77.88	0.49 (0.10)***	0.19	5.17	−11.41 (1.00)***	11.37
IRI-FS	0.13***	48.65	0.21 (0.04)***	0.2	5.22	−3.49 (0.42)***	8.39
IRI-EC	0.08***	28.12	−0.07 (0.04)	−0.07	1.72	−2.94 (0.40)***	7.30
IRI-PT	0.05***	18.04	0.11 (0.04)**	0.11	2.82	−2.22 (0.42)***	5.31
IRI-PD	0.13***	45.72	0.24 (0.04)***	0.24	6.46	−2.76 (0.39)***	7.06

**p < 0.01; ***p < 0.001.

Age and gender account for 23% of the variance in the AES score and for 20% of the IRI total score, with each year of increment in age increasing AES by 0.47 *SD* and by 0.19 *SD. As expected*, being a male decreases all measures of empathy significantly (*p* < 0.001) but with a higher impact on IRI-total (*t* = −11.37, *p* < 0.001). Although age is a significant predictor of IRI total and the FS, PT, and PD subscales, it is not a predictor of EC, which remains stable from pre-adolescence to late adolescence, slightly tending to descend in adults.

As expected, the measures of human and animal empathy were only moderately correlated (*r* = 0.21, *p* < 0.001), and the IRI-Personal distress scale presented the highest correlation among IRI dimensions with AES (*r* = 0.23, *p* < 0.001).

## Study 2. Empathy in adolescents and decoding emotional expression

In this study, we inspected if trait empathy predicts adolescents’ accuracy in emotion identification from emotional expression. It was specifically hypothesized that (H1) the higher the trait empathy (both human-directed and animal directed), the better the accuracy, and that (H2) emotion identification accuracy should be mostly predicted by Empathic Concern and by Perspective-taking (IRI-EC and IRI-PT, respectively), as these are the emotion components that best capture the cognitive and emotional adaptive responses to others’ emotional displays. Skin conductance response (SCR) amplitude was also expected to predict identification accuracy (H3), as it is indicative of an increase in target-related interest and an implicit measure of emotional activation.

Furthermore, we explore a possible association between the subjective evaluation of the stimuli impact (arousal and valence) and the correct identification of emotions.

### Participants

A group of 44 participants (ages 13–18, mean = 15.3, 32 female, and 12 male) that had already been in study 1 volunteered to study 2. They were assessed at their respective schools in a room that was temporarily prepared to be used as a psychology laboratory.

### Measures

#### Subjective appraisal of arousal and valence

Self-assessment report pictoric scales—the *Self-Assessment Manikin* (*SAM*—Bradley and Lang, 1994) arousal scale, and valence scales were used. The Valence scale assesses how positive or negative the emotion one feels in regard to presented stimuli, ranging from extremely unpleasant (1) to extremely pleasant/happy (9). The Arousal scale assesses how excited or apathetic one feels in response to stimuli, and ranges from sleepiness or boredom (1) to extreme excitement (9). Participants rate themselves on these scales as to the perception of how each stimulus makes them feel, following stimulus presentation. Due to their pictoric and schematic nature, these scales overcome language, culture, and age limitations and do not require translation; they are easy to use and enable a prompt response, ideal for the onscreen experiment. The valence and arousal scales have been widely used in emotion studies and their scores have been shown previously to correlate with implicit measures of valence (facial EMG) and arousal (skin conductance, EKG) (e.g., Lang et al., 1993; Bolls et al., 2001).

#### Skin conductance responses

Skin conductance is a measure of reactivity as it reflects changes in the activity of the sympathetic nervous system; while so-called tonic changes, or skin conductance level, reflect more stable and prolonged nuances in that activity, phasic changes in skin conductance, or skin conductance responses (SCRs), reflect reactions to stimuli and are thus considered indicative of emotional responses when one is faced with emotion relevant stimuli (e.g., Öhman et al., 1993). Although skin conductance *per se* does not equate to an emotional response, it is useful to assess, in combination with other measures (self-report such as SAM), whether there is an empathic response and its intensity (e.g., Neumann and Westbury, 2011). In other words, although an SCR does not mean that a vicarious empathic response occurred, the latter will always involve an SCR. And, this is also a non-invasive very comfortable measure for the subject.

#### A measure of emotion identification

Seven options were provided for content attribution – the words happy, angry, sad, surprised, disgusted, neutral, and other appearing on the experiment screen as labels (to choose from by pointing and clicking)—after the presentation of emotional videos containing real-life 4” video clips of expressive behavior in negative, positive, surprise and neutral contexts. The choices were converted into hits/no hits according to their correspondence to the valence in the original context of the video clips; to do this we pooled all the correct identification of negative emotions in a “negative Hit” and that “positive hit” is equivalent to hits in the “happy” category.

### Stimuli, apparatus, and procedures

**FIGURE 3:**
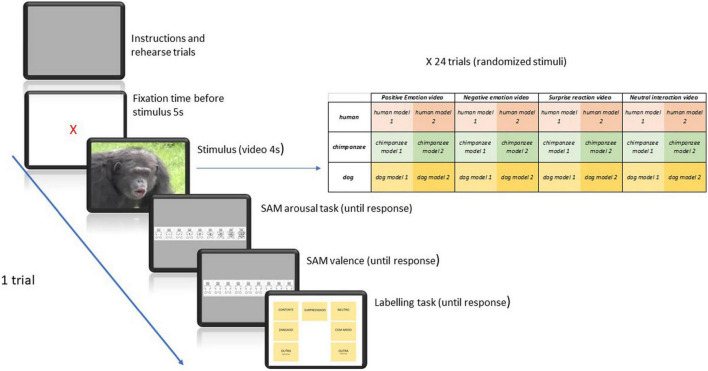
Experimental paradigm in study 2 with the distribution of stimuli and task for each of 24 trials. Stimuli are 4″ video clips. For each emotion category, two different stimuli were presented, with different models (two different humans, chimpanzees and dogs). SAM pictures in the diagram were based on Bradley and Lang (1994) research and the adapted versions herein presented were obtained and used with permission from The Center for the Study of Emotion and Attention, University of Florida.

Stimuli were 24 dynamic pictures, comprising 4” video clips, extracted from original emotional events recorded in emotion eliciting experiments, as part of another project (e.g., Esteves et al., 2015; Gaspar, 2021). The collection included 4 emotion conditions (Happy, Angry, Surprised, Neutral), in three different species (human, chimpanzee, dog), with two different models per emotion, in order to control physiognomic effects, distributed as seen in Figure 3, and presented randomly during the experiments. The videos were close ups of an individual expressive behavior and contained no cues on the context of the behavior.

Subjects were tested individually facing a desktop computer with a 19″ screen. The experimental protocol consisted in viewing a 4 s video clip, then appraising the clip at 3 levels: valence, arousal, and emotion label; for valence and arousal, *SAM* scales were presented onscreen. This was repeated so that each subject observed and rated 6 pictures per emotion, from a total of four emotions, in which three different target species were displayed, with two different individuals representing each target species. Pictures were presented in random order. Exposure time and stimuli order were controlled by an *E-Prime 2* program. Figure 3 illustrates the experimental paradigm.

Skin conductance was recorded simultaneously on a *Biopac* MP100 amplification system, using silver-silver chloride electrodes filled with isotonic electrode paste, attached with adhesive collars to the medial phalanges of the second and third fingers of the subject’s right hand. SCRs were determined by responses with a minimum of 0.05 microSiemens (mS) and latency of 1–4 s after stimulus onset. SCR data were transformed using Lykken and Venables’s (1971) correction and further averaged within each stimulus category *per* participant. All other measures (arousal, valence, and hits) were averaged within participants across presentations of stimuli belonging to the same emotion category.

In this article, we chose to analyze responses and identification of the emotions and not specify differences in identification according to species, as our main focus is, on the one side, empathy, and on the other, the easiness with which differently valenced emotions can be identified. We have previously proposed that positive emotions are easier to identify and are recognized efficiently earlier in life than other emotions because their associated behavior is more stereotypical and predictable (Gaspar et al., 2014), whilst neutral faces are susceptible to labeling that is highly affected by context and observer variables such as culture and expectations (e.g., Jack et al., 2012; Hess et al., 2016). It is expectable that Empathy facilitates overcoming emotion identification problems inherent to negative faces (and possibly, surprise faces as well).

## Results

**FIGURE 4:**
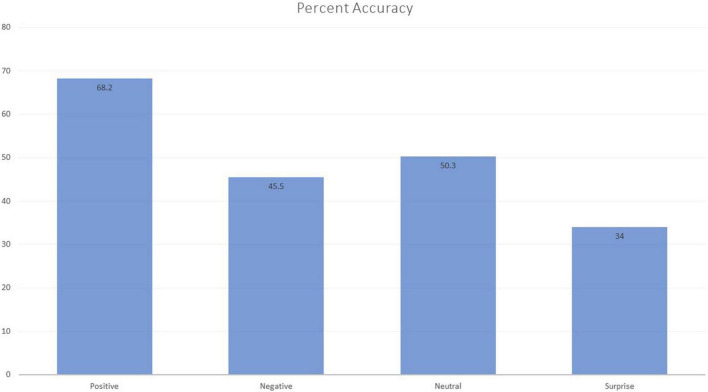
Percent correct identifications in the four presented emotions.

In order to test the recognition of the different emotional stimuli, a 4 × 2 ANOVA was run, with gender as the between-subject variable and emotion as a repeated measure. A main effect of emotion was obtained, *F*(3, 126) 17.01, *p* < 0.001, η^2^ = 0.22, and post-doc analysis (Bonferroni) showed that positive stimuli were significantly more efficiently decoded than the other three categories, which were not significantly differentiated (see Figure 4). Both the main effect of gender, *F*(1, 42) = 0.31, η^2^ = 0.002, and the interaction *F*(3, 126) = 1.13, η^2^ = 0.014, were not statistically significant.

A correlation analysis was run to inspect whether trait empathy correlated with emotion recognition in all four emotion stimuli conditions (H1)—and yielded mixed results, as IRI-total correlated with the correct ID of negative stimuli (*r* = 0.30, *p* < 0.05) but no such association was found with AES (Table 3).

**TABLE 3:** Bivariate correlations between variables in study 2.

Variable	1	2	3	4	5	6	7	8	9	10	11	12	13	14	15	16	17	18
1. HitsPOSIT (1–0)	–																	
2. HitsNEGAT (1–0)	0.13	–																
3. HitsNEUT (1–0)	–0.11	0.03	–															
4. HitsSURPR (1–0)	–0.03	0.22	0.15	–														
5. SCR_POSIT	0.34*	–0.19	0.04	0.13	–													
6. SCR_NEG	0.15	0.19	0.02	–0.03	0.03	–												
7. SCR_NEUT	0.09	–0.05	0.20	0.07	0.16	0.18	–											
8. SCR_SURPR	–0.19	–0.12	0.18	–0.04	–0.02	0.18	2E–04	–										
9. ValencePOSIT	0.37*	0.12	–0.2	0.14	0.27	0.08	–0.13	–0.07	–									
10. ValenceNEG	0.02	–0.10	0.13	–0.9	0.11	0.01	–0.15	–0.06	0.478***	–								
11. ValenceNEUT	0.31*	0.00	–0.11	0.11	0.29*	–0.02	–0.17	–0.21	0.697***	0.514***	–							
12. ValenceSURPR	0.18	0.02	–0.12	0.24	0.22	–0.07	–0.24	–0.18	0.727***	0.618***	0.701***	–						
13. ArousalPOSIT	0.11	0.06	−0.49***	0.05	0.14	0.07	–0.18	0.07	0.407**	0.17	0.27	0.371*	– -					
14. Arousal NEGAT	0.03	0.03	−0.43**	0.05	0.20	0.01	–0.10	–0.16	0.383**	0.23	0.16	0.25	0.799***	–				
15. ArousalNEUT	0.12	0.21	−0.38*	0.09	0.20	0.09	–0.14	–0.04	0.301*	0.18	0.07	0.24	0.795***	0.81***	–			
16. ArousalSURPR	0.03	0.00	−0.45**	0.08	0.24	–0.03	–0.12	–0.18	0.273	0.23	0.16	0.32	0.79***	0.83***	0.89***	–		
17. Iri total	0.13	0.30*	0.16	0.14	0.15	–0.04	–0.01	–0.11	0.518***	0.18	0.528***	0.394**	0.12	0.00	0.10	0.06	–	
18. AES total	0.23	–0.20	–0.12	–0.80	0.12	0.02	0.15	–0.06	–0.175	–0.16	−0.315*	–0.05	–0.08	–0.05	–0.16	–0.08	–0.19	–

*p < 0.05; **p < 0.01; ***p < 0.001.

Contrary to our expectations EC and PT were not correlated with correct emotion identifications, dismissing H2.

In order to test H3, separate regression analyses were conducted for the four emotion categories. Correct recognition of positive stimuli was predicted by subjective valence ratings and SCRs, i.e., higher valence ratings and larger SCRs explained 15.3% of the variance in decoding positive stimuli (*F* = 4.89, *p* = 0.012). Regarding the contributions of the individual predictors, valence was a significant predictor, *t* = 2.07, *p* = 0.045, 0.05, while SCRs were not significant, *t* = 1.69, *p* = 0.10, *b* = 0.46. Regarding negative stimuli, the only significant predictor of correct identification was IRI-total, i.e., higher IRI values could predict 9% of the correct recognition of negative stimuli, *F* = 4.24, *p* = 0.046, *b* = 0.003. No predictors were found for correct identification of the surprise expressions. As to neutral expressions, low arousal ratings could explain 14% of the variance in accurate identification of neutral stimuli, *F* = 6.98, *p* = 0.01, *b* = −0.06.

Interestingly, higher IRI values were correlated with more positive valence evaluation of positive stimuli (*r* = 0.52, *p* < 0.001), surprise (*r* = 0.39, *p* < 0.01), and neutral stimuli (*r* = 0.53 *p* < 0.001), i.e., participants with higher empathy directed to humans evaluated more positively the different stimuli, with the exception of the negative ones.

## General discussion

### The trajectories of adolescent empathy, across targets, and across dimensions

As expected, according to pre-existing literature, self-reported empathy toward humans (Christov-Moore et al., 2014; Rochat, 2022) and toward animals (Gómez-Leal et al., 2021) is more intense in females, and this occurs across all sampled age groups.

The global progression in empathy toward the (higher) adult empathy, especially that of animal-directed empathy and the fantasy dimension of human empathy, seems to nicely fit the patterns of brain development through adolescence by Giedd et al. (1999) and Giedd, 2008—the increase in gray matter in the temporal lobe peaking at 16–17 years old could be related to increased intensity of emotional responses, and the increased pruning and white matter growth in late adolescence might relate, among other things, with the improvement of regulatory processes. But the dimensions of Personal distress, Empathic Concern, and Perspective Taking did not follow a linear growth path.

Empathic concern (EC) did not differ between age groups—and we do not find this puzzling at all: EC’s average was already higher in 11–12-year-old girls than in any other human empathy dimension, showing a trend to a slight increase toward 17–18 years old, only to meet the levels that other dimensions reached in adulthood, which suggests that this dimension of empathy is more intensely downregulated in adulthood. Bearing in mind that EC is the dimension of IRI that best translates the emotional component of empathy, and that emotional empathy has an early development—as compared to cognitive empathy—alongside an earlier and faster maturation of neurological networks, and a consistent gender difference in favor of girls (e.g., Decety and Michalska, 2010; Christov-Moore et al., 2014), the current results not only validate and are validated by those previous findings, but also contribute to establishing that pre-adolescents at 11–12 years old have already in place a strong proneness for emotional resonance to others’ emotions. An important implication of this is that these pre-adolescents hold the potential for important empathy-driven responses, such as compassion and prosocial behavior, and for cognitive appraisals that are affected by the emotional response (such as emotion identification).

To this early development of emotional empathy, interactions within the family contribute immensely, especially those with the mother and/or with primary caregivers (e.g., Hoffman, 1975, 2000; Eisenberg and Strayer, 1987; Schore, 2001). Parental styles throughout childhood and adolescence (Morris et al., 2017) and perceived environment at school also seem to play a role Farrell and Vailantcourt (2021). Notably, the dimensions of mother interaction with the child early in life, such as mother engagement and satisfaction with maternity have been shown, in a 26-year longitudinal study, to predict EC at 31 years of age (Koestner et al., 1990). Genetic predisposition also seems to play a significant role in the development of emotional empathy, as studies with twins reveal a preponderance, continuity and temporal increment of genetic effects (Knafo et al., 2008).

To sum up, emotional empathy understandably does not follow an ascending trajectory from adolescence to adulthood, because it develops earlier and is already high in the transition from pre-adolescence to adolescence. This is a critical information for parents and educators, stressing the relevance of any empathy nurturing programs to begin earlier, especially those that propose to trigger vicariant empathic responses.

Results are also compatible with the possible tendency of increased intensity of negative emotions toward late adolescence and reduced frequency of positive emotions, reported in Bailen et al.’s (2019) review, as these would have the potential to trigger stronger responses. Empathic reactions of negative valence could be potentiated in this age group, something that is not compatible with the personal distress results in this study, that increase toward adulthood—a result that is not easy to explain. It is more likely be that late adolescents (17–18 years old) are only experiencing stronger empathic emotional reactions than their younger peers.

An intriguing finding in S1 was that whilst empathy toward animals increased toward adulthood in both males and females, Empathic Concern and Perspective Taking, which are core dimensions of empathy toward humans, did not. It is conceivable that these transition patterns in human trait empathy toward adulthood are related to increased emotional regulation as EC is concerned, a developmental step that is expected from final processes in brain maturation in regulatory networks Giedd et al. (1999), entwined with culture, which has shown in a variety of studies to strongly influence emotion regulation (Kim and Sasaki, 2012). Adding to their relatively high score at the beginning of adolescence one is left wondering if indeed it is context and culture that generate fluctuations therein. Fluctuations in empathy toward different targets in adolescence have been reported before, but as the targets were either male or female peers, these differences in empathy were interpreted as part of socialization and mating-related bias (Olweus and Endresen, 1998).Which, of course, is compatible with the hypothesis that there is a mediation by context occurring over an empathy trait that is already stabilizing.

Regarding Perspective taking, the differences between males and females are located at 13–14 years old, which is likely to relate to asynchrony in maturation, as girls are ahead of boys in the development of almost all brain structures with volume peaks occurring between 1–3 years earlier in girls (Giedd, 2008), including brain networks central to care and nurturance motivation (Decety and Michalska, 2010; Christov-Moore et al., 2014). The slightly higher trend toward increasing PT in adulthood may simply reflect adjusting to cultural and contextual gender roles and expectations. If empathy and prosociality are more valued in females than males, then PT—the conscious mentalizing—would be expected at least not to decrease in females. Such cultural influence is consistent with the previous finding that adolescents and young adults of different cultures evince significant differences in human trait empathy assessment with IRI (Cassels et al., 2010).

The adult scores in human-directed empathy matched those obtained in other studies with Portuguese samples that used IRI as a measure (e.g., Limpo et al., 2010) and so did adult scores on Empathy toward animals using AES as a measure (Emauz et al., 2016), although AES scores were slightly lower when compared to the average scores of UK adult respondents (Paul, 2000). In regard to Empathy toward animals, previous evidence indicates that it might vary according to beliefs related to the animal’s ability to suffer, experience emotion, and the complexity of the animal mind (Hills, 1995; Knight et al., 2004). And, in that regard, adolescents and adults are exposed to different influences across cultures (e.g., Emauz et al., 2018). This again is compatible with a well-developed empathy trait in pre-adolescents, that is down regulated by mid adolescence and that may, under the circumstance of more information, more exposure to relevant situations is susceptible to increase (but could otherwise have a different path in another culture or context). This remains to be tested of course for animal directed empathy.

So investigating these differentiating factors will inform also the long-standing question of how to promote empathy and specifically bolster empathy toward specific target groups.

### Empathy and decoding emotion expression

Emotion identification results in S2 partially met our expectations: as in previous studies expression of positive emotion was easier to recognize, and, surprise has always been an emotion with a large margin of identification error, even in studies supporting its universality (Ekman et al., 1987). An embodied mechanism such as the vicariant experience in emotional empathy may be an identification facilitator as well, judging from previous research where trait Emotional empathy predicted faster (Besel and Yuille, 2010), stronger and congruent electromyographic responses to perceived facial expressions (e.g., Surakka and Hietanen, 1998; Sonnby-Borgström et al., 2003; Dimberg et al., 2002, 2011) as well as higher accuracy in emotion identification (Dimberg et al., 2011; Rymarczyk et al., 2016), especially to the facial expression of positive affect (Surakka and Hietanen, 1998). EC and full measures of empathy came out as major predictors of emotion decoding in Besel and Yuille’s (2010) study, and under different exposure time conditions, EC provided the best outcome under a nearly subliminal exposure condition.

The fact that the expression of negative emotions was not easily recognized was not entirely surprising, given that only overt (prototypical) expressions of anger and threat tend to be so (Gaspar et al., 2014). But, because the appropriate recognition and label of emotions has been considered a measure of Cognitive Empathy by several authors (Baron-Cohen et al., 2001; Blair, 2005), the absence of a significant correlation between PT and correct identification of both negative and positive emotions was an unexpected finding. In our study the full measure of human-directed empathy (IRI total) was the single predictor of the successful decoding of negative emotion. Previous research using measures of emotional empathy revealed associations between emotional empathy and both implicit and explicit decoding of negative emotion in addition to positive emotion (e.g., Sonnby-Borgström et al., 2003; Rymarczyk et al., 2016), so we think that the emotion identification success in those studies relied in part on using posed, highly stereotyped, non-ambiguous and conspicuous stimuli, whereas our stimuli were none of the above, as they were retrieved from natural behavior recordings, with identified and coded context and emotional interaction, of which our experiment participants had no knowledge.

Although observing others in pain or experiencing negative emotions is a common trigger of empathy in children and adolescents (Hoffman, 1982, 2000; Decety et al., 2008) recognizing emotions and empathizing is largely permeable, at least up to 13 years old, to interactions with parents, including parental emotion expression and regulation (Hoffman, 2000; Bariola et al., 2011) and to the perceived similarities between oneself and the other (Black and Phillips, 1982; Clarke, 1984; Batson et al., 2005) that generate identification and predict empathy (Bloom, 2017). Considering the partial overlap between the neural regions involved in first-hand emotional experience and in recognizing emotion in others (Decety, 2011), especially in painful or distressful situations, the lack of perceived similarity might have been a caveat in our study—our stimuli were varied and encompassed human adult faces, chimpanzee faces and dog faces during emotional interactions—we cannot determine that now and are thus unable to ascertain if lack of identification might have hindered expressive behavior decoding. However, previous research, indicates that negative expressions are, from birth to adulthood more formally diversified and less emotion-specific (e.g., Oster, 2005), and more difficult to decode than those associated with positive emotion (Gaspar et al., 2014; Dúran et al., 2017), and that empathy consistent facial muscle reactions to dog facial emotion expression occur more than to human or chimpanzee facial emotion (Esteves et al., 2015; Gaspar, 2021), sagging that it is more likely that the varied and ambiguous nature of human and chimpanzee facial displays, in the absence of contextual information that critically informs about the emotion, that explains the difficulty in adequate decoding, contrasting to the more stereotyped dog expressions, whose decoding is also facilitated by human familiarity with dogs (Esteves et al., 2015). More research is required to establish if empathy, and specifically, its cognitive dimension (as measured by PT) mediates the ability to overcome these inherent difficulties in decoding negative expressions, in the presence of contextual clues.

de Greck et al. (2012) provide further evidence that culture affects the emotional processing of faces in adults, in a study that, despite finding cross-cultural constants in empathy for anger, also unveiled brain areas where participants of different cultures had different patterns of neural activation during exposure to facial expressions of anger in an intentional empathy task.

Because of such permeability of empathy to environmental influences these findings call for replication and cross-cultural verification.

Perhaps related to positive emotion being easier to identify from expressive behavior than negative emotion, this identification process does not rely as much on empathy—as previously shown psychopaths, characterized by a marked lack of empathy (Hare, 1996), have been reported to perform worse on the identification of negative emotion and differ less from controls in identifying positive emotion (Blair et al., 2001; Marsh and Blair, 2008) although other studies have either reported a more global impairment in expression processing (e.g., Hastings et al., 2008; Pham and Philippot, 2010) or none at all (e.g., Kunecke et al., 2018).

The reason why SCR to positive emotional stimuli and subjective measure of valence predicted decoding of positive emotion may relate to the diversity of the stimuli used—among the positive expression videos some might have been more activating, triggering positive emotion in the participant. The stimuli, by triggering a positive reaction, could have facilitated decoding, through embodiment, which would be predicted by the Perception Action Model. And, it is hard to disentangle what comes first—the facilitating response stemming from one’s experience, or an early predisposition for easier decoding of positive affect than any other that amounts to proposing expression of positive emotion the closest to a prototypical universal, with important social interaction relevance (Gaspar et al., 2014). Although we did not monitor the participant’s facial expression in response to the video stimuli, it is possible that automatic mimicry to positive emotional expression also played a role, as predicted from evidence on the facial feedback hypothesis (Dimberg et al., 2000; Dimberg and Soderkvist, 2011) and by the positive effect of the perceiver’s expressiveness on decoding accuracy (Halberstadt et al., 2011).

That animal-directed empathy did not emerge as a predictor of decoding is intriguing. It is possible that “animal empathizers” direct their attention (and concerns) less to expression and more to context (and our stimuli did not show context). It is also possible that empathy toward animals is more culturally construed than human-directed empathy, as it might depend more on knowledge and beliefs about the animal mind and emotions.

One interesting unexpected result was that IRI total emerged as a predictor of positive valence in all stimuli conditions but the negative expression one. We find this worth further exploring in future studies, as it is in the vein of findings relating empathy to measures of wellbeing, including in adolescence (e.g., Vinayak and Judge, 2018).

### Study limitations and future perspectives

One strength of the current study also generated one of its limitations—the stimuli, which comprised video clips of spontaneous utterances of expressive behavior in emotional situations. By being spontaneous their degree of intensity varied, and their conformity to prototypical facial expressions of discrete emotions did too. In contrast, s*tudies on decoding facial expressions are generally based on highly visible full-blown posed prototypical facial expressions of discrete emotions* (for a discussion of the relevance of using prototypical expression stimuli and the mismatch between real-life expression and posed expressions see Gaspar et al., 2014). Given the *positive effect of the intensity of facial expressions on the accuracy in decoding emotion* (e.g., Halberstadt et al., 2011) with highly intense faces being more accurately decoded than less intense faces, and the greater likelihood that in positive emotion a prototypical face will be displayed (Gaspar and Esteves, 2012; Dúran et al., 2017) it is possible that trait empathy was not a better predictor of surprise because of the absence of a prototypical facial expression of surprise in our stimuli.

In future research, it should be possible to determine whether the increase in Personal Distress toward adulthood is followed by a decrease later on, beyond the twenties, and that was not possible in the current study, due to an unbalanced sample of adults (this sample had an average age of 23.6 years old). The predominant age of our sample is still likely to be a very emotional stage of life with strong development of regulatory processes, and emotion regulation is not yet as well mastered as later on, as suggested by the improved efficacy of regulatory processes in emerging adults when compared to late adolescents (Schweizer et al., 2020). With a more balanced group of adults it would certainly be useful to examine whether splitting adults into further age groups (the twenties, thirties, and so on) would show an increase in some components and a decrease in others—for example reflecting variations on dimensions of empathy, and predictions stemming from them, that may be more dependent on experience.

## Conclusion

This study contributes to understanding the trajectory of empathy from adolescence to adulthood and highlights how pre adolescents enter adolescence already with an head start in emotional empathy. It validates previous studies whereby empathy predicts accurate emotion expression decoding, but only in the case of positive emotions. It breaks with the traditional use of posed expressions to test the empathy-decoding relation, by resorting to naturalistic stimuli. This holds implications for interventions to promote empathy, highlighting the need to increasingly train young participants in being attentive and decoding spontaneous and more varied facial expressions of negative emotion, which represent a bigger challenge.

## Data availability statement

Datasets are available upon request. The raw data supporting the conclusions of this article will be made available by the authors, without undue reservation.

## Ethics statement

The studies involving human participants were reviewed and approved by the board of approval of the Portuguese Government Ministry of Education, and follow the ethical guidelines approved by the author’s research center direction board. Written informed consent to participate in this study was provided by the participants’ legal guardian/next of kin.

## Author contributions

Both authors participated in the conceptualization of the study, data collection, and all stages of manuscript preparation.
